# Aggregates of IVIG or Avastin, but not HSA, modify the response to model innate immune response modulating impurities

**DOI:** 10.1038/s41598-018-29850-4

**Published:** 2018-07-31

**Authors:** Swamy Kumar Polumuri, Lydia A. Haile, Derek D. C. Ireland, Daniela Verthelyi

**Affiliations:** Division of Biotechnology Review and Research-III, Office of Biotechnology Products, Center for Drug Evaluation and Research, Food and Drug Administration, Silver Spring, MD 20993 USA

## Abstract

Therapeutic proteins can induce immune responses that affect their safety and efficacy. Product aggregates and innate immune response modulating impurities (IIRMI) are risk factors of product immunogenicity. In this study, we use Intravenous Immunoglobulin (IVIG), Avastin, and Human Serum Albumin (HSA) to explore whether increased aggregates activate innate immune cells or modify the response to IIRMI. We show that increased aggregates (shaken or stirred) in IVIG and Avastin, but not HSA, induced activation of MAPKs (pp38, pERK and pJNK) and transcription of immune-related genes including IL8, IL6, IL1β, CSF1, CCL2, CCL7, CCL3, CCL24, CXCL2, IRAK1, EGR2, CEBPβ, PPARg and TNFSF15 in human PBMC. The immunomodulatory effect was primarily mediated by FcγR, but not by TLR. Interestingly, increased aggregates in IVIG or Avastin magnified innate immune responses to TLR2/4 agonists, but diminished responses to TLR3/9 agonists. This study shows that IIRMI and aggregates can modify the activity of immune cells potentially modifying the milieu where the products are delivered highlighting the complex interplay of different impurities on product immunogenicity risk. Further, we show that aggregates could modify the sensitivity of PBMC-based assays designed to detect IIRMI. Understanding and managing immunogenicity risk is a critical component of product development and regulation.

## Introduction

Therapeutic proteins and peptides, whether recombinant, synthetic, or naturally derived have the potential to induce an immune response in the host that impacts on the safety and efficacy of the product. Product immunogenicity is not necessarily tied to adverse effects but can neutralize the product’s activity or result in changes in product pharmacokinetic or pharmacodynamics profiles, ultimately affecting the product’s safety or efficacy and depriving patients of important therapies^1^. Occasionally, the immune response can elicit cross-reactive antibodies that neutralize low-expression non-redundant endogenous proteins in the host leading to deficiency syndromes as observed in patients that developed episodes of pure red cell aplasia or thrombocytopenia secondary to the development of antibodies to Eprex® and megakaryocyte growth and development factor (MGDF), respectively^2,3^. Thus, understanding and managing immunogenicity risk is a critical component of product development and regulation.

Currently most therapeutic proteins have low levels of aggregates, however these can form during manufacturing, shipping, and storage. Protein aggregation has been identified as a key risk factor in protein immunogenicity assessments^4–6^. This is supported by clinical data showing reduced immunogenicity in products that changed their manufacturing process and reduced their aggregate content^5,7^. For example, historically patients receiving human growth factor had a high incidence of persistent ADA (50–70%), which was reduced to <10% following a reduction in the aggregate content in the product^7^. Similarly, human gamma-globulin displayed increased immunogenicity as compared to the aggregate-free version^8,9^. More recently, product denaturation and aggregation were linked to the emergence of neutralizing antibodies in a clinical trial for erythropoietin^10^. These clinical observations are supported by several studies in mice showing that aggregated proteins are more immunogenic than monomers^4,6^. Further, aggregates may facilitate breaks in tolerance as suggested by studies where treatments with aggregated human IFNα induced anti-drug antibodies (ADA) in transgenic mice expressing human IFNα^11^. Several mechanisms by which aggregates mediate increased immunogenicity have been proposed, including modification of the pharmacokinetics and tissue distribution of the product, direct crosslinking of B cell receptors, increased uptake of particulate matter by antigen presenting cells (APC), and shifting intracellular trafficking and processing^4,12,13^. However, the mechanism by which aggregates foster immunogenicity are still not well understood^14–16^. This is partly because the effects of aggregates on immunogenicity may differ depending on their composition, morphology, size and charge; which in turn are heavily dependent on the characteristics of the monomer and the conditions under which the aggregates are formed^7,17–21^.

One possible mechanism by which aggregation increases immunogenicity risk involves the induction of local inflammation and activation of innate immune cells embedded in the tissues where the product is administered^22–24^. It is known that protein aggregates can induce local inflammatory responses when deposited on tissues, as reported in patients with Alzheimer’s, where deposits of Beta-amyloid causes local inflammatory responses, tissue damage and an influx of immune cells^25^. This immune activation is thought to be partly linked to the activation of innate immune receptors on immune cells and the local release of pro-inflammatory cytokines^26^. Among therapeutic proteins, aggregates of monoclonal antibodies were shown to crosslink Fcγ receptors (FcγR) on the surface of B cells, dendritic cells, macrophages and neutrophils embedded in the tissues with greater affinity than monomers^27^ and trigger the activation of innate effector cells. This enhances antigen presentation and maturation of dendritic cells (DCs)^28^. Further, recent studies suggest that aggregates of monoclonal antibodies (mAbs) induce an inflammatory response by PBMC that is not evident when monomers are used. Indeed, blocking of FcγR I, FcγR II and FcγRIII reduced the interaction between aggregated mAbs and FcγRs reducing the pro-inflammatory effect induced by antibody aggregates^27,29^. Lastly, recent reports suggest that Human PBMC, human monocytes and THP-1 cells could interact with Toll-like receptor (TLR) 2 and TLR4 to produce cytokines and chemokines when stimulated *in-vitro* with aggregated proteins^29,30^.

Toll-like Receptors are one of several families of germline-encoded pattern recognition receptors (PRRs) expressed on sentinel cells such as macrophages and dendritic cells that recognize structurally conserved molecules derived from microbes^31^. Their ligands include various bacterial cell wall components such as lipopolysaccharide (LPS), peptidoglycan (PGN) and lipopeptides, as well as flagellin, bacterial DNA and viral RNA. Also, a variety of intracellular proteins such as heat shock proteins as well as protein fragments from the extracellular matrix can trigger them. These receptors initiate signaling cascades leading to the activation of transcription factors, such as AP1, NFκB and interferon regulatory factors (IRFs). In recent years, our group and others have suggested that trace levels of process or host cell derived impurities, or contaminants introduced unintentionally during the manufacturing process can activate PRR increasing the risk of product immunogenicity^22,32–37^. Further, several studies suggest that multiple innate immune response modulating impurities (IIRMI) can act on different receptors or cell types synergizing their effect on local inflammation and systemic immune activation, making IIRMI an important risk factor for immunogenicity^32,38–40^. In this study, we characterize the pro-inflammatory activity of protein aggregates and determine whether they synergize with different IIRMI to increase the immunogenicity risk of therapeutic proteins. We show that aggregates of IVIG, which is a complex mixture of human immunoglobulins, or Avastin, a monoclonal antibody (mAb) targeting VEGF-A, can modulate the response to IIRMI magnifying the induction of IL8, IL1β, and IL6 in response to TLR2 or TLR4 agonists. Interestingly, the same aggregates reduced the CXCL10, ISG15 and MX1 response to TLR9 (CpG) or TLR3 (poly I:C) agonists. The effect of the aggregates of antibodies was not universal as aggregates of HSA induced minimal innate immune activation and did not modulate the pro-inflammatory activity of endotoxin. Our study results highlight the interplay between product-related risk factors and the complexity of predicting immunogenicity risk.

## Material and Methods

### Drug products

Recombinant Human serum albumin (HSA) therapeutic grade (Albucult, Novozymes, Nottingham, UK), IVIG (Gammagard; LE12L036AC, LE120389AB, LE12R014AB; Baxter Healthcare Corporation, Westlake Village, CA) and Avastin (Bevacizumab, lot 3063923 and 3022680; Genentech Inc. South San Francisco, CA) were used in these studies. Purified PRRagonists (PRRAgs) including lipopolysaccharide (LPS, TLR4), Pam3Cys4, (P3C; TLR2), and poly I:C, (TLR3) were obtained from InvivoGen (San Diego, CA, USA). TLR9 agonists CpG ODN D-35, (5′-ggTGCATCGATGCAGGGGgg-3′) and CpG ODN B2006 (TCGTCGTTTTGTCGTTTTGCTGTT) were synthesized at the FDA core facility (Rockville, MD, USA).

### Mice

C57BL/6 (WT) and MyD88^−/−^ mice were housed in sterile microisolator cages under 12-hour day/night cycle and given food and water ad libitum in the specific pathogen-free, AAALAC accredited animal facility of the U.S. Food and Drug Administration’s Division of Veterinary Medicine (Silver Spring, MD). This study was carried out in strict accordance with the recommendations in the Public Health Service Policy on Humane care and Use of Laboratory Animals. All protocols involving animals were approved by the Animal Care and Use Committee at US-FDA.

### Cell culture and *in vitro* stimulation

Unidentified PBMC (5 × 10^6^cells/well) obtained from the NIH blood bank were cultured in 24 well plates using RPMI (FCS 10%, glutamate (2 mM), Penicillin-Streptomycin (50 μg/mL) 10 mM HEPES, B-mercaptoethanol (1 µl /mL) and stimulated with aggregated or unaggregated protein (80 μg/mL as described in^30^). In some studies, various concentrations of purified PRRagonists (PRRAgs) including lipopolysaccharide (LPS, TLR4), Pam3Cys4, (P3C; TLR2), CpG ODN and poly I:C, (TLR3) were added for the time intervals indicated in the individual figure legends.

Bone marrow derived macrophages (BMDM) were obtained from WT C57BL/6, FcγR^−/−^ (kindly provided by Dr. Jeff Ravetch, Rockefeller University, USA), and MyD88^−/−^ mice. BMDMs were cultured in DMEM supplemented with 20% (vol/vol) LADMAC (ATCC) conditioned medium as source of macrophages colony stimulating factor-1, 10%FBS, 2 mM glutamate, penicillin and streptomycin (50 μg/mL) for six days. Cells were washed and stimulated with aggregated or unaggregated IVIG either in the presence or absence of LPS (10 ng/mL) for 3 h in the absence of conditioned medium.

### Preparation and characterization of protein aggregates

Recombinant protein HSA, IVIG and Avastin were diluted to 1 mg/mL in sodium citrate buffer (10 mM sodium citrate, 5% sucrose, pH 6.0) and used either as 1) unaggregated protein (room temperature for 20 h), 2) shaken protein (450 rpm for 20 h at 25 °C), or 3) stirred protein (using glass vial with a Teflon stirrer at 1100 rpm at room temperature for 20 h). Aggregated and unaggregated proteins were kept refrigerated at 4 °C until further use and were stable in solution for up to 30 days. All preparations tested negative by LAL for endotoxin.

### Optical density

The optical density of aggregated and unaggregated HSA, IVIG and Avastin was measured using the Agilent Carry 100 UV-VIS spectrophotometer (Agilent Technologies, Santa Clara, CA).

### SDS-PAGE analysis

Aggregated and unaggregated proteins (10 µg) were loaded on Bio-Rad Mini-PROTEAN TGX, 4–20% gels (Hercules, CA) in the presence or absence of reducing agent. For reducing condition aggregated or unaggregated protein were treated with Laemmli lysis buffer with a reducing agent (Fermentas/Thermos Fisher Scientific, Waltham, Massachusetts, USA) at 100 °C for 5 minutes. Electrophoresis was carried out and gels were stained with Coomassie Brilliant Blue and photographed.

### FlowCAM

A FlowCAM PV-100 bench top system (Fluid Imaging technologies, Scarborough, ME) fitted with an FC100 flow cell, a 10X objective, and collimator, and a 0.5 mL syringe. The gain and flash duration were set such that the average intensity means of the image was consistently between 180 and 200. Initial calibration was carried out by flushing five times with 1 mL water. The aggregated and unaggregated IVIG, Avastin and HSA samples were analyzed at a flow rate of 0.145 mL/min without dilution. Particles ranging in size (2 μm–2 mm) (equivalent spherical diameter) were counted and normalized by dividing the number of particles by the total volume imaged for each sample to obtain the particle concentration (#/mL). In addition to the samples, buffer solutions were also analyzed by FlowCAM. The data is shown after subtraction of particles from sample buffer.

### Micro Flow Imaging (MFI)

The number and size of particles in aggregated and unaggregated IVIG, Avastin and HSA were measured using the MFI 5200 system from Proteinsimple (San Jose, California, USA) equipped with a 100-micron flow cell and MFI view system software, (version 2-R2.6.1.20.1915). The system was cleaned with purified distilled water at maximum flow rate with the plush mode. Flow cell cleanliness was visibly checked confirmed visually before running the samples. The samples were analyzed at a flow rate of 0.17 mL/mL and fixed camera. Prior to use, the MFI performance was calibrated using 5 μm particles/mL (Duke Standards, ThermoFisher Scientific, Fremont, CA) and NIST traceable size standard 3000 particles, (ThermoFisher Scientific, Fremont, CA). The samples were diluted 1000X in sodium citrate buffer. For each read 0.9 mL of product was prepared. The purge volume was 0.2 mL and analyzed sample volume was 0.6 mL. The MFI was set to capture 20,000 images/sample. Samples were measured in triplicate. The data is shown after subtraction of particles from sample buffer as background.

### NFkB-activation assays

HEK293 cells that co-express stably individual human TLR 2,4,5, or 9 and an NF-κB/AP-1-inducible secreted embryonic alkaline phosphatase (SEAP) reporter gene (InvivoGen, San Diego, CA) were cultured in DMEM (10% FCS with 50 μg/mL penicillin-streptomycin, 100 μg/mL normocin, 2 mM L-glutamine supplemented with 1X HEK-BLUE Selection) and supplemented with Blasticidin and Zeocin as per manufacturer’s instructions. The cells were plated at InvivoGen’s recommended density in 100 µL in flat bottom 96-well plates and stimulated with 100 µL of aggregated or unaggregated protein (80 µg/mL) or TLR ligands for 24 h. Supernatants were collected and NFkB activation was measured using detection medium with quanti-blue preparation per the manufacture’s protocol. Briefly, 150 µL of quanti-blue were added per well in a 96 well flat bottom with 50 µL of supernatant from the stimulated samples with unaggregated product, aggregates or TLR ligands (positive controls). After 2 h incubation at 37 °C, the SEAP levels were determined colorimetrically at 620 nm by spectroscopy.

### FcγR blocking

Human PBMC (5 × 10^6^cells) were plated per well in 24 well plate in the presence of FcγR cocktail containing of F(ab)2 anti- FcγRI(anti-CD64, clone 10.1), F(ab)2 anti- FcγR II (anti-CD32, clone 7.3), F(ab)2 anti- FcγR III (anti-CD16, clone 3G8) (each 10 µg/mL) and isotype control (10 µg/mL) (Ancell, Bayport, MN, USA), incubated for an hour at 37 °C before stimulation with aggregated or unaggregated protein (80 µg/mL) for 24 h. mRNA of CCL2, CCL7, PPARG, EGR2, SPP1 and IL10 were measured by real-time PCR and protein in 24 h supernatants by using a Luminex ProcartaPlex® Immunoassay Kit (eBioscience) as per the manufacture’s recommendations.

### mRNA isolation and measurement by quantitative real-time PCR

Total RNA was isolated using Trizol reagent from Invitrogen (Carlsbad, CA, USA) as specified by the manufacturer. RNA was quantified by spectrophotometric analysis using the Nanodrop (ThermoFisher Scientific, Wilmington, DE). The cDNA was prepared from 1 μg of total RNA using high capacity cDNA Reverse Transcription Kit (Applied Biosystems, Foster City, CA) as per the manufacturer’s recommendation. Real-time PCR using TaqMan probes (Applied Biosystems, Foster city, CA) was conducted using the Viia7 Real-time PCR system. The change in the relative expression levels of mRNA was calculated by normalizing against house-keeping genes and are expressed as fold increase over media treated cells using the 2^−ΔΔCt^ method^41^.

### Nanostring mRNA Profiling

The NanoString nCounter Human Immunology V2 Panel (NanoString Technologies, Seattle, WA) was used in these studies per manufacturer’s instructions. Briefly, probes were hybridized to 100 ng of total RNA for 19 h at 65 °C, after which excess capture and reporter probes were removed, and transcript-specific ternary complexes were immobilized on a streptavidin-coated cartridge. The code set contains a 3′ biotinylated capture probe and a 5′reporter probe tagged with a fluorescent barcode, two sequence-specific probes for each of 594 transcripts. All solution manipulations were carried out using the NanoString preparation station robotic fluids handling platform. Data collection was carried out with the nCounter Digital Analyzer to count individual fluorescent barcodes and quantify target RNA molecules present in each sample. Normalization was performed based on a standard curve constructed using the spike in exogenous control samples. Background hybridization signal was determined using the spike in negative controls provided. mRNAs with counts lower than the mean background +2 standard deviations were considered to be below the limits of detection. The nSolver (v.2.5) user interface (Nanostring) was used to operate the nCounter Advanced analysis module, which employs the R statistical software. The global gene expression data is presented in volcano plots, which show each target’s p value (log_10_) and fold change (log_2_) relative to unstimulated (M) cells. Highly significant targets are displayed on the top of the graph and highly differentially expressed genes fall to either side. Green point colors and horizontal lines indicate the various false discovery rates. The differences in expression for individual genes were tested by ANOVA (Graph pad Prism 7.0). The datasets generated during and/or analyzed during the current study are available from the corresponding author on reasonable request.

### Western blot analysis

Human PBMC were washed with PBS and then lysed in buffer (Cell Signaling, Beverly, MA) with protease inhibitor cocktail (Roche), and boiled for 5 min with Laemmle’s lysis buffer for SDS-PAGE. Twenty µg of total protein were run on a Bio-Rad Mini-PROTEAN TGX, 4–20% gradient gel in Tris/glycine/SDS buffer (25 mM Tris, 250 mM glycine, 0.1% SDS) (Bio-Rad,Hercules, CA), and then electrotransferred onto Immobilon-P transfer membranes (Millipore, Bedford, MA) at 100 V for 2 h (4 °C). After blocking for 1 h in TBS-T (20 mM Tris-HCl, 150 mM NaCl, 0.1% Tween 20) containing 5% nonfat milk, membranes were washed 3 times in TBS-T and probed for 20 h at 4 °C with anti-pp38, anti-pERK, anti-pJNK, anti-pAKT, anti-pSTAT1 (701), anti-IRF7 and anti-β-actin (Cell signaling, Beverly, MA, USA) per manufacturer’s instructions. Following washing in TBS-T, membranes were incubated with secondary HRP-conjugated, anti-rabbit IgG or anti-mouse IgG from Cell Signaling (1: 2,000 dilution) for 1 h at room temperature.The bands were detected using ECL plus reagents (ThermoFisher Scientific, Rockford, USA) and quantitated with ImageJ software.

### Cytokine Quantitation

Cytokine were measured in 24 h supernatants using a Luminex ProcartaPlex® Immunoassay Kit from the eBioscience as per the manufacturer’s recommendations.

### Statistical analysis and software

Statistical comparisons were assessed using a corrected multiple T test or one-way or two-way ANOVA followed by Bonferroni’s multiple comparisons test as appropriate. Statistical analyses were performed with Graph Pad Prism 7.0 Statistical Software (Graph Pad Software Inc., San Diego, CA, USA). Synergy: a regression modelling cytokine production as a function of TLR ligand concentration, product aggregation, and their interaction was used to assess whether product aggregation and TLR ligand concentration affect cytokine production and whether such contribution was synergistic. A significant interaction between the two main effects would denote a synergistic association. This regression was fit using the PROC GLM procedure in SAS®, version 9.4. Statistical significance was defined as *P* < 0.05.

## Results

### IVIG aggregated protein induced innate immune response in human PBMC

Aggregated therapeutic proteins are more immunogenic than their non-aggregated counterparts but the precise underlying mechanisms remain unclear^4,6,30,42^. Recent reports suggest that the increased immunogenicity could be mediated in part by aggregates inducing pro-inflammatory cytokines and chemokines^32,43^. To better understand the immunomodulatory effect of aggregated proteins, we induced the formation of aggregates in intravenous immunoglobulin (IVIG) by shaking or stirring the product for 20 h at room temperature as previously described^29,30^. IVIG is comprised primarily of polyclonal gamma globulins (IgGs) isolated and pooled from the plasma of healthy donors and is currently used to treat several autoimmune diseases^44^. The increased level of aggregates was evidenced by augmented absorbance at wave length ranges between 320–400 nm using light scattering analysis^45,46^ (Supplementary Fig. 1A). The size of the aggregates was modified under reducing conditions indicating the presence of disulfide bonds (Supplementary Fig. 1B). Characterization of the size distribution of the aggregates formed using FlowCam and MFI shows significantly increased (p < 0.001) number of particulates of all sizes ranging from 1–100 µm but particularly sub-visible ones (Supplementary Fig. 2). Of note, although all the methods showed that stirring and shaking increased the content of IVIG aggregates, the absorbance, SDS PAGE and MFI results suggested that the stirred preparation appeared to have relatively higher number of particles, particularly smaller than 10 μm, compared to the shaken samples. While this difference was not evident when using FlowCam, small differences between methods used to characterize aggregates are not unexpected^47^.

**Figure 1:**
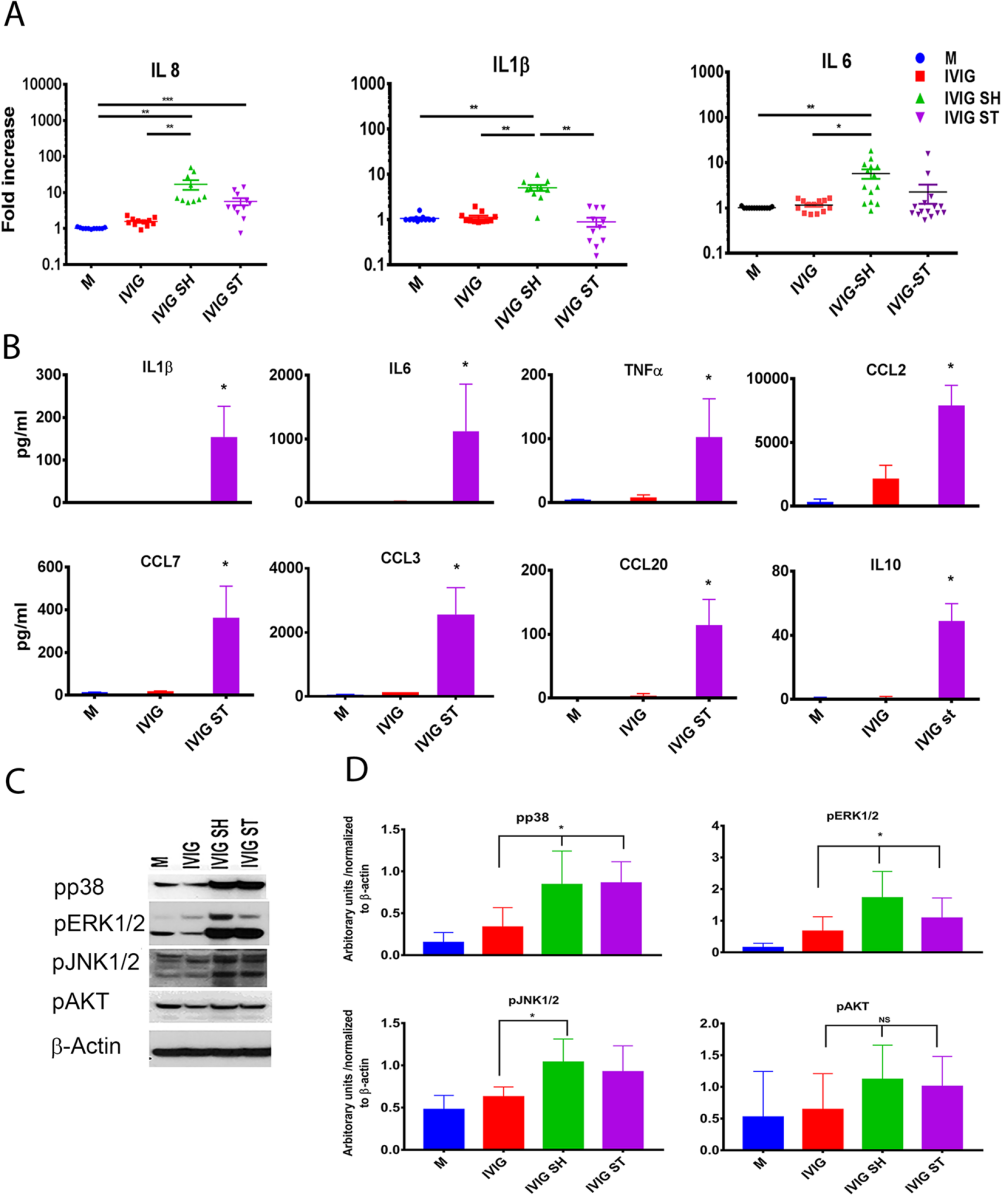
Induction of pro-inflammatory cytokines and MAP Kinase activation in human PBMC by aggregated IVIG: (**A**) The mRNA of IL8, IL1β and IL-6 was measured by quantitative real-time PCR after stimulation for 24 h with IVIG, or IVIG subjected to shaking (IVIG SH) or stirring (IVIG ST) for 20 h (80 µg/mL). Unstimulated cells were used as negative controls (denoted as M). Dots represent individual blood donors. Fold expression is calculated over media treated cells. (**B**) Levels of IL1β, IL6, TNFα, CCL2, CCL7, CCL3, CCL20 and IL10 in supernatant of PBMC stimulated with IVIG or aggregated IVIG for 24 h were determined by Luminex. Data shows mean ± SEM derived from the n = 3 donors. *p < 0.05. (**C**) Whole cell lysates were prepared after stimulation with intact  or aggregated IVIG for 30 minutes, and then subjected to Western blot analysis using antibodies to pp38, pERK1/2, pJNK1/2, pAKT and β-actin. One of three experiments with similar results. (**D**) Mean densitometry data was quantified after normalization to β-actin.

Using these stressed products, we next determined the effect of increased IVIG aggregates on the expression of genes linked to innate immune activation and inflammation. Stimulation of human PBMC with aggregated IVIG protein, particularly those prepared by shaking, resulted in increased transcripts for IL1β, IL6, and IL8 as compared to PBMC from the same donors cultured in the presence of unaggregated IVIG (Fig. 1A). The increased inflammatory response was also evident in the increased levels of pro-inflammatory cytokines (IL1β, IL6, TNFα and IL10) and chemokines (CCL2, CCL7, CCL3, CCL20) in the 24 h cell supernatants (Fig. 1B). Further, the increase in pro-inflammatory cytokines was associated with increased activation of map kinases- pp38, pERK1/2 and pJNK, which were evident within 30 minutes of stimulation of PBMC with protein aggregates (Fig. 1C,D). This suggested that the aggregated IVIG activates innate immune responses directly.

**Figure 2:**
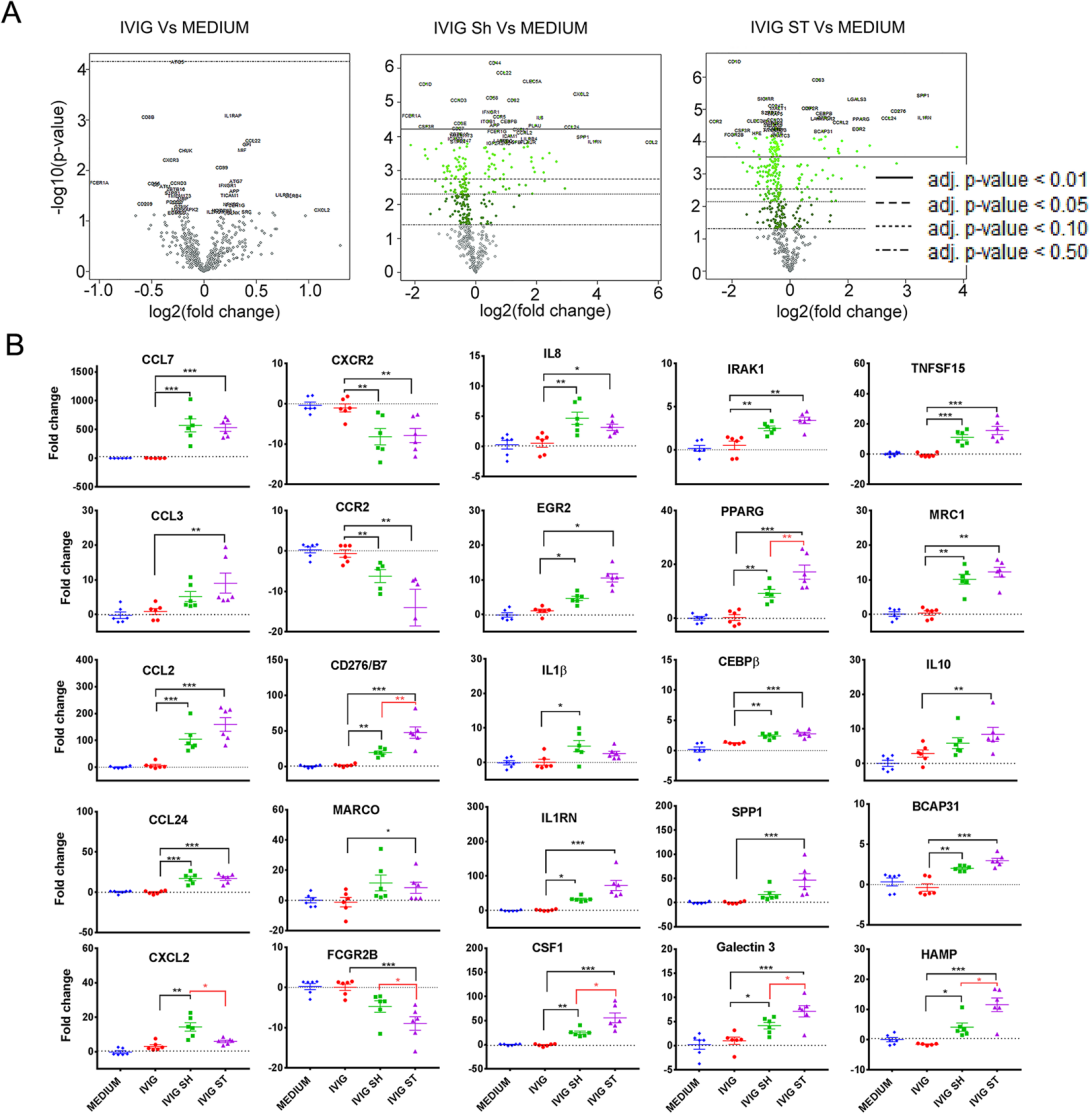
Increased aggregate levels in IVIG activate innate immune responses in PBMC: (**A**) Volcano plot showing mRNA expression levels in PBMC (n = 6) stimulated *in vitro* for 24 h with undisturbed or aggregated IVIG as determined by using NanoString nCounter with Human Immunology V2 Panel. Volcano plots generated using the nSolver 3.0 program. Green point colors and horizontal lines indicate the various false discovery rates. (**B**) Fold change in mRNA expression for CCL7, CXCR2, IL8, IRAK1, TNFSF15, CCL3, CCR2, EGR2, PPARG, MRC1, CD276/B7, IL1β, CEBPβ, IL10, CCL24, MARCO, IL1RN, SPP1, BCAP31, CXCL2, FCGR2B, CSF1, GALECTIN3 and HAMP after stimulation with intact or aggregated IVIG for 24 h. Each dot represents a different donor (n = 6). Fold change was calculated relative to the corresponding mean gene expression in untreated cells. Mean and SEM are noted. All genes identified showed statistical difference between groups by nonparametric ANOVA. Black lines and asterisks denote significance in post-analysis multiple comparison. Red lines denote significant differences as determined by a t-test between cells exposed to stirred or shaken IVIG. * < 0.05, ** < 0.01, *** < 0.001. Unstimulated cells were used as negative controls (denoted as Medium).

To obtain a more complete characterization of the effects of aggregates on the immune response, we next stimulated PBMC with unperturbed or aggregated IVIG for 24 hours and screened gene expression using a Nanostring Immunology panel of 594 genes linked to the immune response. Results showed that unperturbed IVIG did not induce a significant increase in gene expression, whereas the same cells stimulated with IVIG containing increased aggregates modified the expression of over 100 genes (Fig. 2A and Supplementary Table 1). Salient among the responses is the increased expression of multiple genes linked to the recruitment, maturation, and activation of monocyte/macrophages and neutrophils including CCL7 (MCP3), CCL2 (MCP1), CCL3 (MIP1a), CCL24 (eotaxin-2), and CXCL2 (MIP2a) as well as the downregulation of chemokine receptors CCR2 and CXCR2 (Fig. 2B)^48,49^. Increased expression of CD276/B7 and MARCO and reduced FCGR2B (FcγRIIb) also suggest the activation of macrophages. In addition, the IVIG aggregates increased the expression of pro-inflammatory signals including IL8, IL1α and IL1β, as well as markers linked to innate immune activation via TLR such as serine/threonine kinases intermediate molecule IRAK1 and transcription factor CEBPB, TNFSF15 and HAMP^50^. Of note however, the increase in IL1RN, CSF1, Galectin 3, PPARG, EGR2, and reduction of CXCR2 also suggest an anti-inflammatory or regulatory macrophage component to the response^51^.

Interestingly, although the expression of most genes was similarly modified by shaken and stirred products, PBMC stimulated with stirred IVIG had relatively higher transcripts for PPARG, CD276/B7, IL-1RN, CSF1, and EGR2, hepcidine (HAMP) and lower CXCL2, IL-8, IL-6 and IL-1b (Figs 1A and 2B). Together this suggests that aggregates in IVIG can modulate the immune milieu fostering an immune response.

### Innate immunity activated by IVIG aggregates is partially mediated by FcγRs

**Figure 3:**
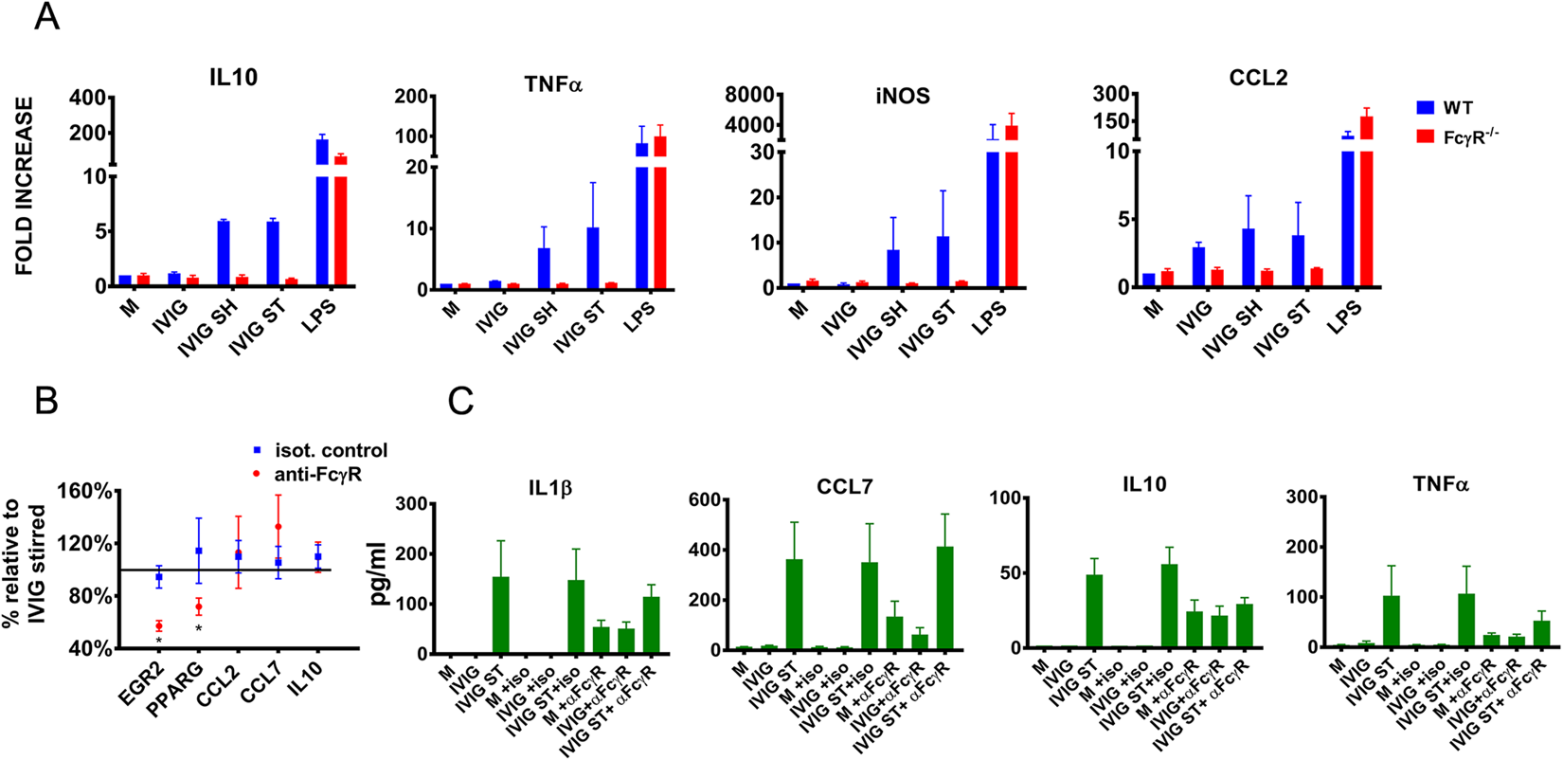
Role of FcγRs in innate immune response to aggregated IVIG: (**A**) Cytokine expression in bone marrow-derived macrophages from WT and FcγR^−/−^ stimulated with intact or aggregated IVIG (80 µg/mL). Positive control: LPS (10 ng/mL). Results shown as mean ± SEM for 2 independent experiments with 3 mice each. B&C) Cytokine expression by PBMC stimulated with aggregated IVIG in presence or absence of anti-FcγRs antibody cocktail containing (FcγR I, FcγR II, and FcγR III, each 10 µg/mL) or the corresponding isotype controls for 24 h (n = 4). (**B**) Shows changes in mRNA expression relative to aggregated (stirred) IVIG in cells that were pre-treated with the anti-FcγRs cocktail relative to those that were not exposed to the cocktail for the same subject. (**C**) Shows protein concertation in 24 h supernatants as analyzed by Luminex. Unstimulated cells were used as negative controls (denoted as M).

IVIG is used in the treatment of several pro-inflammatory diseases and the underlying mechanism is thought to be linked to the blocking of FcγR on the surface of B cells and monocytes^52^. Previous studies suggested that aggregated immunoglobulins cross-link FcγR on the surface of monocytes more efficiently than monomeric ones eliciting an inflammatory response^29^ and blocking of FcγR was shown to reduce the secretion of pro-inflammatory cytokines by human PBMC and THP-1 cells stimulated with aggregated mAbs^29,30^. To determine whether FcγR mediates the increase in cytokine levels induced by aggregated IVIG, we stimulated BMDM from C57BL6 or FcγR^−/−^ mice. As shown in Fig. 3A, the IVIG preparation enriched in aggregates did not induce expression of pro-inflammatory genes in macrophages from FcγR^−/−^ mice suggesting that in mice FcγR mediate the response to aggregates. Several reports have suggested that the role of FcγR may differ in mice and human, therefore we next exposed PBMC to a cocktail containing neutralizing concentrations of F(ab)2 anti- FcγR 1, F(ab)2 anti-FcγR II, F(ab)2 anti- FcγR III prior to stimulating the cells with unaggregated or aggregated IVIG. As shown in Fig. 3B, the anti-FcγR cocktail reduced the level of expression of PPARG and EGR2 mRNA in human PBMC stimulated with IVIG aggregates. However, it did not modify the CCL2 and CCL7 mRNA levels as compared to those induced in the presence of isotype controls. Similarly, as shown in Fig. 3C, pre-incubation of PBMC with the anti-FcγR cocktail reduced the levels of IL-10 and TNFα but did not significantly reduce the levels of IL1β, and CCL7 secreted into the supernatants. Of note, these results must be considered carefully as addition of the anti-FcγR cocktail induced a pro-inflammatory response that was independent of the presence of IVIG aggregates (Fig. 3C). Together, these data suggest that FcγR partially mediate the pro-inflammatory effect of aggregates, but other mechanisms of cellular activation are involved.

### Role of TLR in innate immune activation by IVIG aggregates

**Figure 4:**
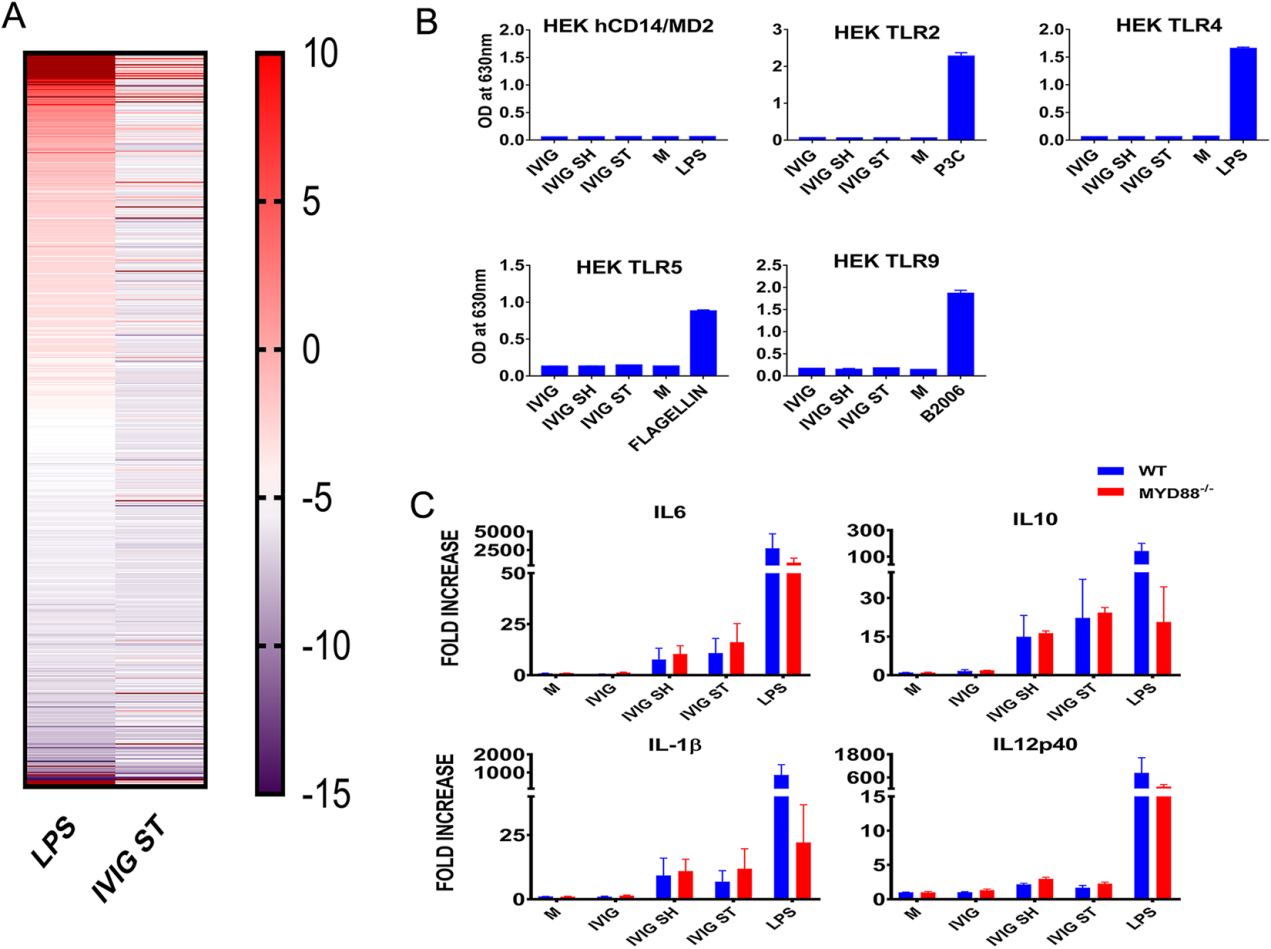
Role of TLR in Innate immune activation by IVIG aggregates (**A**). RNA expression as assessed by NanoString nCounter using a Human Immunology V2 Panel. PBMC were stimulated *in vitro* for 24 h with LPS (100 pg/mL) or IVIG containing increased concentration of aggregates due to stirring. (**B**) Stimulation of HEK-blue cells expressing individual TLR with IVIG. HEK-blue –hTLR2 (5 × 10^4^), HEK-hTLR 4 (2.5 × 10^4^), HEK-hTLR5 (2.5 × 10^4^), HEK-TLR9 (8 × 10^4^) and HEK-hCD14/MD2 (5 × 10^4^) were stimulated with aggregated or unaggregated IVIG (80 µg/mL) or with respective positive controls for 24 h. The level of NFκB activation in the supernatant was measured using QUATI-BLUE as described in materials and methods. (**C**) Response of BMDMs of MyD88 KO mice to aggregated and unaggregated IVIG. Expression of IL6, IL10, IL1β and IL12p40 mRNA by bone marrow derived macrophages of age-matched C57Bl/6 or MyD88^−/−^ mice as determined by RT-PCR. Cells were stimulated with intact, shaken or stirred IVIG (80 µg/mL) for 24 hours. Cells stimulated with trace levels of LPS (10 ng/mL) were used as positive controls. Results shown as mean + SEM of 2 independent experiments with 3 mice each. Unstimulated cells were used as negative controls (denoted as M).

Recent studies suggest that aggregates can activate innate immune responses *in vitro* by directly stimulating TLR2 and TLR4 in human monocytes or PBMC^29,43,53^. These receptors activate cells via NF-κB leading to the production of pro-inflammatory IL1β, IL6 and IL-8. We reasoned that if aggregates were activating PBMC primarily via TLR4, then they would induce a similar pattern of gene expression as the canonical TLR4 ligand, LPS. Therefore, we first compared the response of PBMC from 6 blood donors cultured in the presence of very low levels of endotoxin (100 pg/mL), unperturbed, or aggregated IVIG (Fig. 4A). IVIG aggregates and endotoxin induced several common genes, however the patterns of gene expression were clearly distinct. For example, at 24 hours, IVIG aggregates-induced high levels of CSF1, CCL7, CCL2, CL24, PPARG, SPP1, EGR2, MRC1, LGALS3, HAMP, BCAP31, and G6PD while endotoxin-induced relatively higher levels of IL1α, IL1β, IL6, IL8, and IL10. This was not surprising as protein aggregate would be expected to engage multiple receptors, including complement and scavenger receptors. To further explore a role for TLRs we next used the unperturbed and aggregated IVIG to stimulate HEK293 cells that co-express individual TLR gene and an NF-κB/AP1-inducible *SEAP* (secreted embryonic alkaline phosphatase) reporter gene. Previous studies had shown HEK293 cells expressing TLR2 to be activated by soluble aggregates of amyloid peptides^54^. As shown in Fig. 4B, the IVIG aggregates failed to activate HEK293 cells expressing TLR2, TLR4, TLR5 or TLR9). Since it was possible that aggregates required multiple TLR receptors, such as TLR2-TLR6 heterodimers, or a co-factor to induce a pro-inflammatory response, we next compared the response to aggregated IVIG by BMDM from wild type C57BL/6 mice and mice lacking MyD88 (MyD88^−/−^), a universal adaptor protein required for signaling by almost all TLR except TLR3. As shown in Fig. 4C, while MyD88^−/−^ mice had impaired response to LPS, there were no differences in mRNA levels of IL6, IL10, IL1β and IL12p40 in BMDM from MyD88^−/−^ after stimulation with IVIG aggregated protein. Together, this indicates that IVIG aggregates do not require TLRs or Myd88 to induce a pro-inflammatory response.

### Innate immune response to aggregates in the presence of trace levels of TLR2/4 agonists

**Figure 5:**
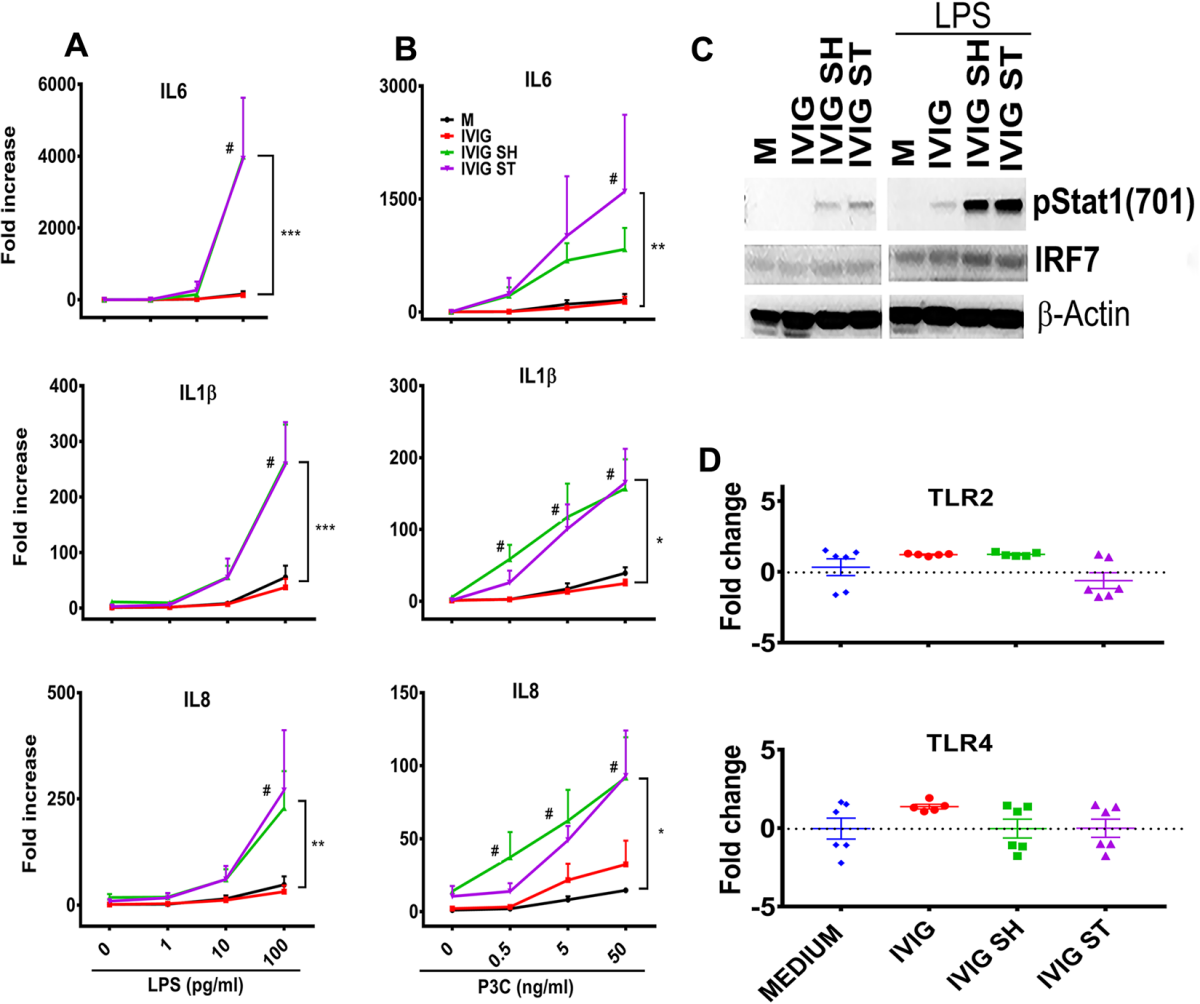
Trace levels of TLR2 and TLR4 agonists and IVIG aggregates synergize to augment the innate immune response. (**A**,**B**) mRNA of IL6, IL1β and IL8 were measured by real-time PCR in PBMC stimulated with intact or aggregated IVIG (80 µg/mL) in the absence or presence of increasing concentrations of LPS (**A**;1 to 100 pg/mL) or Pam3Cys4 (**B**; 0.5 ng to 50 ng/mL). Results shown represent 2–3 independent experiments with 1–3 subjects each. Differences in gene expression were tested by two way ANOVA # < 0.05. Synergy testing: Significant interactions in the effect of TLR agonist and aggregation were tested using SAS^®^9.4 as described in the materials and methods section and are denoted with *, where * < 0.05, ** < 0.01, and *** < 0.001. (**C**) STAT1 phosphorylation and IRF7 expression were measured in whole cell lysates of PBMC stimulated with intact or aggregated IVIG in the presence or absence of LPS (100 pg/mL for 12 h) by Western blot. Results are representative of 2 independent experiments with healthy blood donors. (**D**) TLR2 and TLR4 mRNA expression in Human PBMC after stimulation with aggregated or unaggregated proteins of IVIG, for 24 h was analyzed by Nanostring. Data shown as fold change in expression over the mean expression in unstimulated cells. Unstimulated cells were used as negative controls (denoted as Medium).

*In vitro* and *in vivo* studies with mouse and primate models have shown that trace levels of innate immune response modulating impurities (IIRMI) can induce the expression of pro-inflammatory cytokines such as IL1β, IL6, and IL8^55,56^. Such an increase in inflammatory cytokines is associated with enhanced antigen presentation and was shown to correlate with increased risk of immunogenicity for therapeutic proteins *in vivo*^22,57^. Since both IIRMI and aggregates could be present in a therapeutic protein preparation, we next explored whether the pro-inflammatory effect of aggregated IVIG would modify the response to trace levels of impurities. As expected, incubating PBMC with increasing concentration of LPS (1 pg to 100 pg/mL) induced IL6, IL1β and IL8 mRNA expression in a dose dependent manner (Fig. 5A). The addition of unperturbed IVIG did not modify the levels of LPS-induced IL6, IL1β and IL8 mRNA. In contrast, the addition of aggregated IVIG significantly increased mRNA for IL6, IL1β and IL8 at every concentration of LPS indicating that the two types of impurities could synergize to increase the risk of immune activation. Furthermore, as shown in Fig. 5C and Supplementary Fig. 3A, the phosphorylation of pSTAT1 and total IRF7 were increased in protein lysates of samples stimulated with shaken or stirred IVIG in the presence of LPS (100 pg/mL). Of note, this low level of LPS (100 pg/mL) induced a modest increase in pSTAT1, but did not induce detectable increases the activation of pERK1/2, pJNK1/2, pp38, and pAKT in PBMC regardless of the presence of aggregates (Supplementary Fig. 3B). Interestingly, the increase in pSTAT1 or cytokine mRNA does not appear to be associated with the upregulation of TLR4 as the mRNA expression is not modified by aggregated IVIG (Fig. 5D). To determine whether the synergistic effect observed was selective for endotoxin, we next explored whether aggregates of IVIG modified the response to TLR2 agonist P3C. PBMC stimulated with P3C (0.5 ng to 50 ng/mL) showed increased levels of IL6, IL1β and IL8 mRNA in a dose dependent manner (Fig. 5B). Addition of aggregated IVIG (shaken or stirred) further increased the expression of these genes, whereas similar levels of unperturbed IVIG failed to do so. As above, the increased response was not associated with an increased expression of TLR2 mRNA. Despite this, the IVIG aggregates magnified the response to trace levels of TLR2 and TLR4 ligands potentially increasing the immunogenicity risk of trace levels of IIRMI.

### Impact of aggregates on the immunomodulatory effect of nucleic acids

**Figure 6:**
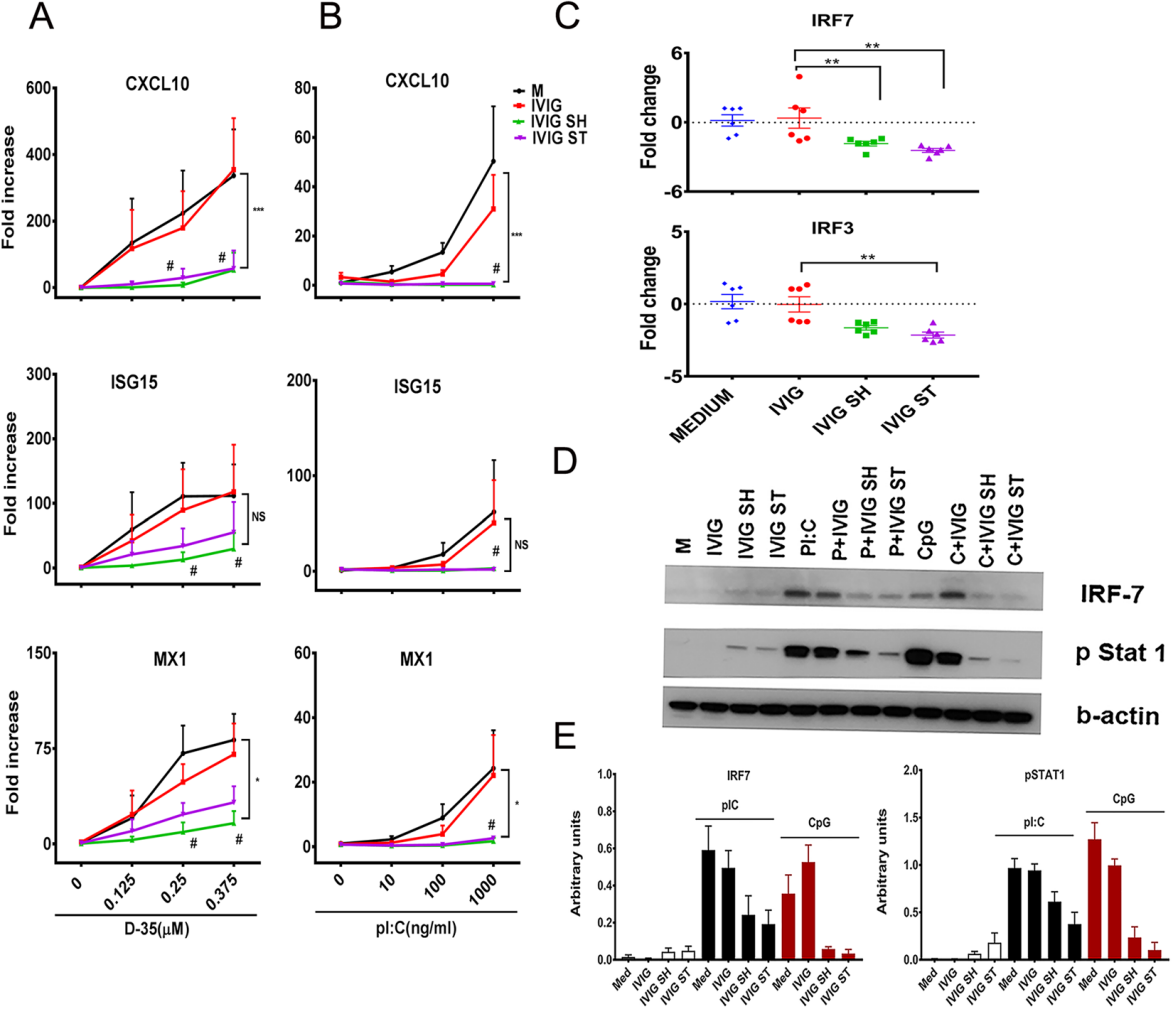
IVIG aggregates reduce the IFN response to TLR3 and TL9 agonists. (**A**,**B**) mRNA levels for CXCL10, ISG15 and MX1 were measured by RT-PCR in PBMC stimulated for 24 h with intact or aggregated IVIG (80 µg/mL) in the presence of increasing concentrations of a TLR9 ((A) CpG ODN D-35, 0.125 μM to 0.375 μM) or a TLR3 ((**B**) Poly I:C, 10 ng to 1 μg/mL) agonist. Results shown represent 3 independent experiments with 1–3 donors each. Differences in gene expression were tested by two-way ANOVA # < 0.05. Synergy testing: Significant interactions in the effect of TLR agonist and aggregation were tested using SAS^®^9.4 as described in the materials and methods section and are denoted with *, where * < 0.05, ** < 0.01, and *** < 0.001. (**C**) TLR7 and IRF3 mRNA in Hu-PBMC stimulated with intact or aggregated IVIG for 24 h as assessed by Nanostring. Each dot represents an individual blood donor. (**D**) Levels of pStat1 and IRF7 were measured by Western blot in whole cell lysates of Hu-PBMC stimulated for 12 h with intact or aggregated IVIG in the presence CpG ODN D-35 (0.375 μM) or PolyI:C (1 μg/mL). (**E**) The densitometry data from the Western blot was quantified and normalized to β-actin. Data shows mean ± SD of 2 independent experiments. Unstimulated cells were used as negative controls (denoted as M or Med. or Medium).

Biologics can contain nucleic acids derived from the host cell or adventitious agents that are present during manufacture. Multiple receptors have been described in the endosomes and cytoplasm that recognize nucleic acids and can foster an immune response^58^. To determine whether IVIG aggregates would also increase the response to nucleic acids, we next assessed the response to TLR3 agonist Poly I:C and TLR9 agonist CpG ODN in the presence or absence of IVIG aggregates. These receptors mediate the production and phosphorylation of IRF3 and IRF7 leading to the secretion of type 1 interferons and IFN stimulated genes (ISG); thus CXCL10, ISG15 and MX1 were used to monitor their activity. As shown in Fig. 6, PBMC stimulated with CpG ODN (Fig. 6A) or Poly I:C (Fig. 6B) increased the mRNA level for ISGs in a concentration dependent manner. The addition of unperturbed IVIG did not modify the response; however, the addition of aggregated IVIG to the culture significantly reduced the mRNA levels for the ISGs. The reduction in the IFN response was associated with lower levels of mRNA for IRF7 and IRF3, (Fig. 6C) as well as lower levels of pSTAT1 and IRF7 relative to those induced after stimulation with CpG ODN or Poly I:C alone (Fig. 6D,E). Of note, while CpG ODN exerts most of its activity via IRF7, it does induce some activation of NFkB that can be gauged by assessing IL-8 and IL-6 expression. As shown in Supplementary Fig. 4A, the presence of aggregates did not modify the levels of IL-6 or IL-8 induced by CpG ODN. Similarly, there were no changes in pERK1/2, pJNK1/2, pp38, and pAKT in PBMC stimulated with CpG ODN alone or together with the IVIG aggregates (Supplementary Fig. 4B). Therefore, the presence of aggregated IVIG can differentially regulate the biological activity of IIRMI.

### Aggregates of mAb Avastin but not HSA modify the innate immune response to IIRMI

**Figure 7:**
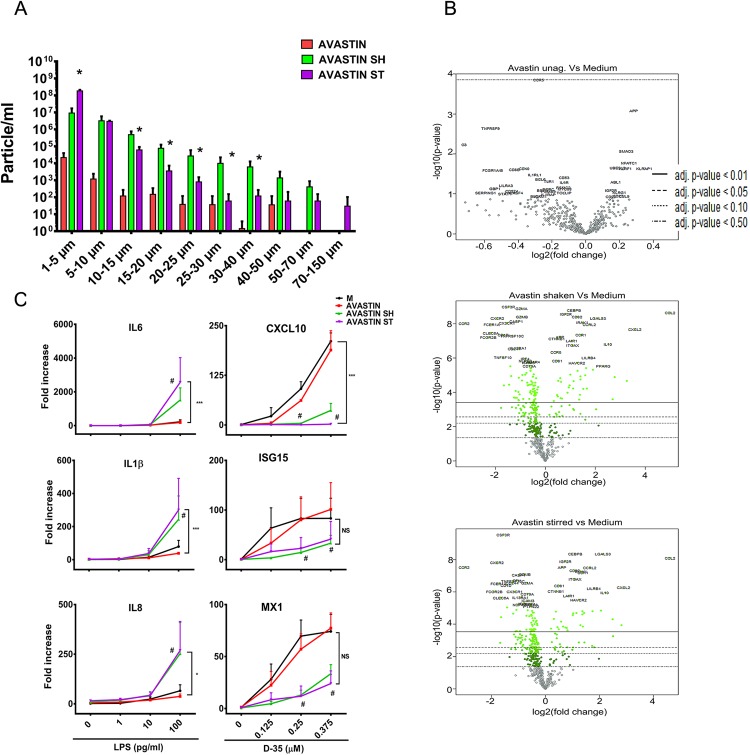
Aggregates of Avastin synergize with trace levels of TLR2 and TLR4 agonists. (**A**) Shaking and stirring aggregates Avastin. Shaking or stirring of Avastin (1 mg/mL in sodium citrate buffer; 10 mM sodium citrate, 5% sucrose and adjusted the pH to 6.0) leads to the formation of particles as determined changes to the MFI. (**B**) Volcano plot showing mRNA expression levels in PBMC (n = 6) stimulated *in vitro* for 24 h with undisturbed or aggregated Avastin (80 μg/mL) as determined by using NanoString nCounter with Human Immunology V2 Panel. Volcano plots generated using the nSolver 3.0 program. Green point colors and horizontal lines indicate the various false discovery rates. (**C**) Expression of pro-inflammatory genes following the stimulation of Hu-PBMC (mean + SEM of 3 donors) with aggregates of Avastin alone or together with increasing levels of LPS (1 ng to 100 ng/mL) or CpG ODN D35 (0.125 μM to 0.375 μM). Differences in gene expression were tested by two way ANOVA # < 0.05. Synergy: Significant interactions between TLR agonist and aggregation were tested using SAS as described in the materials and methods section and are denoted with *, where * < 0.05, ** < 0.01, and *** < 0.001. Unstimulated cells were used as negative controls (denoted as M or Medium).

IVIG is a complex biologic product comprised primarily of immunoglobulins of different specificities and previous studies had suggested that it can downregulate TLR responses, although the mechanism is unknown^59,60^. To determine whether the pro-inflammatory effect of aggregates and the magnification of the effect of IIRMI was restricted to IVIG we produced aggregates of Avastin, a humanized recombinant monoclonal mAb that binds VEGF-A. As shown in Fig. 7A, shaken and stirred Avastin tend to form fewer and smaller aggregates compared to IVIG under similar conditions. Despite this, they induced a similar pattern of gene expression as the aggregated IVIG with increased expression of chemokines CCL2, CCL7, CXCL2, CXCL13, and IL8, as well as several markers linked to macrophage activation including IL10, C3, CD276, SPP1, PPARG, MARCO, IL1RN, IRAK1, and antimicrobial peptides such as Galectin 3, CCL8, and HAMP (Fig. 7B and Supplementary Fig. 5). Moreover, the presence of product aggregates magnified the response to low levels of LPS (TLR4 ligand) while reducing the response to CpG ODN (TLR9 ligand) (Fig. 7C). This supports the conclusion that the presence of aggregates in antibody therapeutics can increase the risk of inducing a local innate immune response and could magnify the risk posed by some IIRMI present in the product. It also suggests that immunoglobulin aggregates may mask the presence of impurities that stimulate some innate immune receptors such as TLR3 or TLR9 in cell based assays.

**Figure 8:**
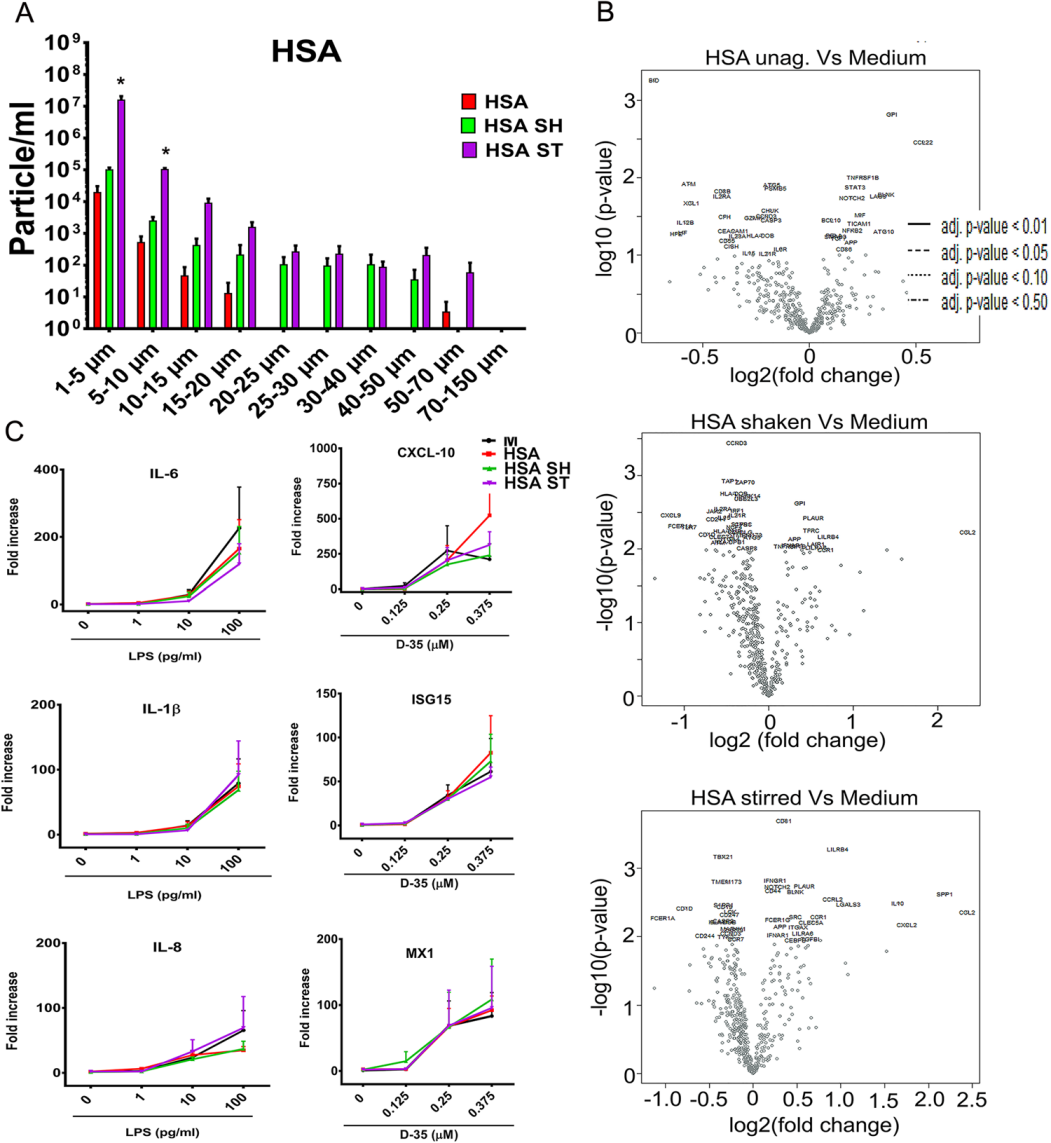
Increased aggregates in HSA do not induce inflammation or synergize with trace levels of TLR2 or TLR4 agonists. (**A**) HSA aggregates formed by shaking or stirring leads to the formation of particles as determined changes to the MFI. (**B**) Volcano plot showing mRNA expression levels in PBMC (n = 6) stimulated *in vitro* for 24 h with undisturbed or aggregated HSA (80 μg/mL) as determined by using NanoString nCounter with Human Immunology V2 Panel. (**C**) Expression of pro-inflammatory genes following the stimulation of Hu-PBMC (mean + SEM of 3 donors) with HSA aggregates alone or together with increasing levels of LPS (1 ng to 100 ng/mL) or CpG ODN D35 (0.125 μM to 0.375 μM). Unstimulated cells were used as negative controls (denoted as M or Medium).

To explore whether the immunomodulatory effect of aggregates extended to other therapeutic proteins, we studied the effects of aggregated HSA. Human serum albumin is frequently used in the formulation of biologics. The aggregates formed by HSA were smaller and fewer than those formed under the same conditions by IVIG or Avastin (Fig. 8A) and failed to induce the expression of innate immune and inflammation related genes (Fig. 8B and Supplementary Table 2). Further, the presence of HSA aggregates did not modify the response to LPS or CpG ODN indicating that not all protein aggregates have a direct immunomodulatory effect (Fig. 8C). These data is consistent with FcγRs playing an important role in the induction of inflammation by aggregates and underscores the role of the underlying structure of the protein in the immunomodulatory effect of the aggregates they form.

## Discussion

Immunogenicity risk cannot be predicted from protein structure alone as it is influenced by a myriad of often interacting parameters such as product structure, preexisting tolerance, immunological status, HLA type, and underlying disease. Two product-related factors thought to be critical in determining whether a product will be immunogenic are the presence of product aggregates and IIRMI such as residual host DNA, host cell proteins, endotoxins and other microbial fragments^32,61^. Separately these different types of impurities have been shown to act as unintended adjuvants that can activate immune cells directly, mediate immune cell recruitment, enhance antigen uptake and processing, and foster adaptive immune responses. In these studies, we sought to characterize better the pro-inflammatory response induced by aggregates and determine whether their presence could modify the response to trace levels of IIRMI, thus increasing the immunogenicity risk of a therapeutic protein. Our results show that increased aggregate content in IVIG or Avastin leads to the induction of a complex innate immune response characterized by increased activation of p38, JNK and ERK, and consequent IL1β, IL6 and IL8 suggesting a pro-inflammatory response that could increase the immunogenicity risk. The innate immune activation induced by immunogloblin aggregates is partially dependent on FcγRs but not does not appear to require TLR2 or TLR4 receptors unlike what had been previously suggested^29,43^ since the absence of MyD88 did not modify mRNA expression in response to the aggregates. Further, we show that the presence of IVIG aggregates magnified the expression of pro-inflammatory cytokines in response to TLR2 and TLR4 agonists, whereas they reduced the response to nucleic acid impurities that stimulate a type I IFN responses, indicating that the interplay between aggregates and IIRMI is complex. Together these data suggest that protein aggregates and some IIRMI can synergize to activate pro-inflammatory responses and increase the immunogenicity risk for therapeutic products and underscores the complexity of assessing immunogenicity risk.

The formation of product aggregates can take many forms depending on the structure of the product and the conditions that facilitate the aggregation. In this study, we have singled out aggregates formed by physical stress (stirring or shaking conditions). While these conditions are thought to represent conditions that model stress during distribution and storage of the product, the concentration of particles used are beyond what would be typically found in commercial products and accounts for a worst-case scenario used to explore any potential impact of such aggregates in a physiologically relevant system. In our studies, increased levels of antibody aggregation led to the induction of pro-inflammatory genes. In contrast, despite forming aggregates of similar sizes and binding FcRN^62^, HSA induced minimal innate immune activation in PBMC and did not modulate the pro-inflammatory activity of endotoxin or CpG ODN. This shows that the underlying structure of the aggregated product plays a critical role in its ability to activate innate immune responses. This is consistent with previous studies showing increased binding of aggregated IgA or IgG to FcγR as compared to monomers^63,64^ as well as studies in neutrophils showing that crosslinking of FcγRs with aggregated IgG leads to p38, ERK and JNK phosphorylation, activation of NFkB and subsequent induction of proinflammatory cytokines^63–65^. Surprisingly, while FcγR were absolutely required for activating mouse BMDM, blocking FcγRs in PBMC with a combination of anti-FcγRs monoclonal antibodies (previously shown to reduce signaling via FcγR^29^) only partially reduced the transcription of IL10 and TNFα, and not that of CCL3, CCL7, or IL1β. This could be due to achieving only partial neutralization of the receptors with the concentrations used, or to biological differences between human and mouse cells. Of note, as shown in Fig. 3C and previously reported^29^, the available neutralizing antibodies to FcγR can stimulate PBMC leading to increased IL1β, IL10 and TNFα levels even in the absence of aggregated monoclonal antibody^29^. This suggests that these results must be interpreted cautiously as the pro-inflammatory response to the cocktail may have masked a reduction in response to the FcγRs mediated activation by the IVIG aggregates. Additional studies will need to address whether different isotypes of aggregated Ig or Fcγ fusion proteins preferentially bind FcγR resulting in various downstream signaling and activation.

Regarding the role of TLR in innate immune stimulation by IVIG, previous studies that used anti-TLR antibodies to block the pro-inflammatory response of aggregated mAbs suggested that TLRs play a key role in the innate immune response to aggregates^30^. Surprisingly, in our studies HEK-293 expressing individual TLRs failed to be activated by the aggregates. Further, BMDC from MyD88 KO mice induced similar cytokine levels as those from the parental strain suggesting that TLR responses are not required for the response of aggregated IVIG (Fig. 4). There are several possible reasons for the disparity in results. First, it is possible that biophysical differences between the aggregates used in the studies published and those generated in our labs or differences in experimental conditions may underlie the difference in the results. For example, previous studies have shown that soluble aggregates of islet amyloid polypeptides stimulate HEK293 expressing TLR2 cells, whereas fibrillar ones do not^54^. While we characterized our aggregates regarding their size distribution, at this time we do not have a good understanding of the specific characteristics of protein aggregate that are critical to induce NFκB activation. In addition, the difference in results may be rooted in minor differences in cellular distribution and TLR gene sequences between humans and mice that can modify the regulation and activation of TLRs. Moreover, while MyD88 is generally recognized as the key mediator for all toll receptors except for TLR3, MAL and TRIF are also adaptor proteins for TLR and could facilitate a TLR4 mediated response. Lastly, innate immune cells are armed with a variety of PRR including Fc, complement and scavenger receptors, that may compensate for the absence of TLR. Importantly, regardless of the receptors involved, it is clear that product aggregation can foster innate immune modulation impacting on the immunogenicity risk of therapeutic antibodies. As we improve our characterizations of the quality attributes of aggregates, and gain a better understanding of their specific effect on innate immune cells we will be able to elucidate what types of aggregates are more likely to increase the immunogenicity risk or interfere with an assessment of process related impurities.

The crosstalk between TLRs and FcγR has been previously described, although the mechanisms underlying it are still unclear^32,38,39^. TLR signal primarily via MyD88, TIRAP, and TRAF6, which ultimately leads to the activation of transcription factors such as nuclear factor kappa B (NF-κB) and activator protein 1 (AP-1). In contrast, FcγR lead to recruitment of tyrosine kinase Syk leading to increased calcium flux and NADPH activation. Although we did not see an increase in TLR expression in the presence of aggregates, we did see increased levels of IRAK1, CEBPB, TNFSF15 and HAMP which could indicate increased signaling. Further, TLR and FcγR can be found in the same lipid rafts and TLR4 was shown to activate Syk directly in neutrophils and macrophages^66,67^, and both FcγR and TLRs were shown to downregulate miRNAs that regulate the production of pro-inflammatory cytokines IL-6 and TNFa^66–68^. Therefore, the possibility that crosslinking of FcγR by immunoglobulin aggregates may augment TLR2/4 mediated activation is not unexpected. In contrast, the signaling that leads to a reduced response to TLR9 and TLR3 agonists in the presence of IVIG aggregates is less clear. However, previous studies have shown that NFkB driven maturation of pDC may led to reduced type I IFN production, and a recent study suggests that immune complexes may downregulate TLR9 via FcγRIIb^69^. Lastly, although these studies explored the role of FcγR and some TLRs, there are multiple other receptors that may be involved in the pro-inflammatory response observed *in vitro*. Understanding these interactions will require further studies, but the observation clearly illustrates the complexity of signals that could be elicited by product and process related impurities that stimulate multiple receptors and underscores the difficulty of predicting immunogenicity risk.

Previous studies had identified IL6, IL8 and IL1β as biomarkers of innate immune response activation by aggregates^22,29,30^. Our studies confirm their findings, but provide a more detailed gene expression analysis of the *in vitro* response that suggests that small structural differences in the aggregates formed by different stresses can result in different patterns of innate immune activation. Indeed, as shown in Figs 2, 7, and 8, the response to aggregates varied depending on the characteristics of the forming monomer and the stress applied, with aggregates formed by stirring inducing higher levels of markers associated with M2 activation of macrophages (MRC1, EGR2, PPARG, IL10, IL-1RN, CCL24, CCL18), a finding that will require further investigation^70–72^. Importantly, in these studies we used PBMC as the platform to explore the response to aggregates. While PBMC are frequently used to assess for innate immune modulators, the frequency of neutrophils, and particularly macrophages and dendritic cells is very low, suggesting that the immunomodulatory effects in the tissues, where there is greater frequency of FcγR and PRR rich cells may be more pronounced. Detailed studies linking the physicochemical properties of the aggregates induced by different stressors to the kinetics of the pro-inflammatory response may be helpful in fully understanding the response to aggregates.

Although all proteins, self and foreign, bear antigenic sites to which an immune response can theoretically be directed, the actual development of an immune response depends on numerous host and product-related factors. One of the critical risk elements for immunogenicity is the presence of impurities of bacterial or host cell origin that can act as adjuvants to the product. These impurities are recognized by several families of PRR that are engaged in surveillance of the extracellular and intracellular space for the presence of microbial products or endogenously derived danger signals. Each of these receptors is triggered by unique microbial signatures but evokes mostly overlapping responses that are primarily channeled through the activation of NFkB and AP1, and result in the production of pro-inflammatory cytokines (IL6, TNFα, IL1β) and chemokines (CXCL8/IL8, CCL5, CCL7 and CCL2). Numerous studies have shown that multiple ligands can activate PRR. Indeed, over 20 different ligands have been identified for TLR4^73^. Since it is not possible to predict the complete array of possible impurities that could be present in a product, we have previously suggested using cells bearing multiple PRRs to determine whether the sum of impurities present in a product could trigger an inflammatory response that would increase the likelihood of an immune response to inform the immunogenicity risk^55^. The results presented herein suggest that the presence of aggregates may magnify the response to some impurities while dampening the response to others. When used together with TLR2 or TLR4 agonists, the enhancement was not mediated by increased TLR expression and did not seem to modify the minimal concentration of PRRAgs capable of inducing a detectable response in PBMC; however, the expression level of proinflammatory cytokines and chemokines was significantly increased as compared to that of cells exposed to the same PRRAgs in the presence of the unaggregated product. In contrast, in the presence of aggregates PBMCs required higher levels of TLR3 or TLR9 agonists to become activated, which could make cell-based assay to detect IIRMI less sensitive to these types of impurities. Interestingly the reduction was evident for genes linked to the activation of IRF7, IRF3, and STAT1, but not for the expression of IL6 or IL8, which is mediated via NFkB activation suggesting that the aggregates specifically reduced the IRF-IFN path^32,38^. Importantly, since different stresses stimulate distinct mechanisms of aggregation and result in different types of aggregates, from soluble oligomers to high molecular weight particles, it will be important to understand what type and level of aggregates can be tolerated when using cell-based assays to assess IIRMI. Future identification of biomarkers of innate immune activation that can differentiate between stimulation by aggregates or IIRMI will help to evaluate and mitigate the risk posed by innate immune activation.

## Electronic supplementary material

Supplementary information
