# Supplementary material for: Aggregates of IVIG or Avastin, but not HSA, modify the response to model innate immune response modulating impurities
Source: Sci Rep. 2018 Jul 31;8:11477. doi: 10.1038/s41598-018-29850-4 (PMC6068171; doi:10.1038/s41598-018-29850-4)
Supplement: Supplementary file 1 — Supplementary information [file 41598_2018_29850_MOESM1_ESM.pdf]

Aggregates of IVIG or Avastin, but not HSA, modify the response to model innate immune response modulating impurities

Swamy Kumar Polumuri, Lydia A. Haile, Derek D.C. Ireland, and Daniela Verthelyi*

DBRR III, OBP, OPQ, CDER, FDA

Division of Biotechnology Review and Research-III, Office of Biotechnology Products, Center for Drug Evaluation and Research, Food and Drug Administration, Silver Spring, MD 20993.

Supplementary Table 1: Distribution of differential genes expression after stimulation with aggregated or unaggregated protein of IVIG in human PBMC

Adj. p-value(<0.05) genes	M vs IVIG un	M vs IVIG sh	M vs IVIG st
Log2 fold change (+ve)	0	63	50
Log2 fold change (-ve)	0	56	144

Supplementary Table 2: Distribution of differential genes expression after stimulation with aggregated or unaggregated protein of Avastin or HSA in human PBMC

Adj. p-value(<0.05)genes	M vs Avastin un	M vs Avastin sh	M vs Avastin st
Log2 fold change (+ve)	0	60	52
Log2 fold change (-ve)	0	131	125
**Adj. p-value(<0.05) genes**	**M vs HSA un**	**M vs HSA sh**	**M vs HSA st**
Log2 fold change (+ve)	0	0	0
Log2 fold change (-ve)	0	0	0

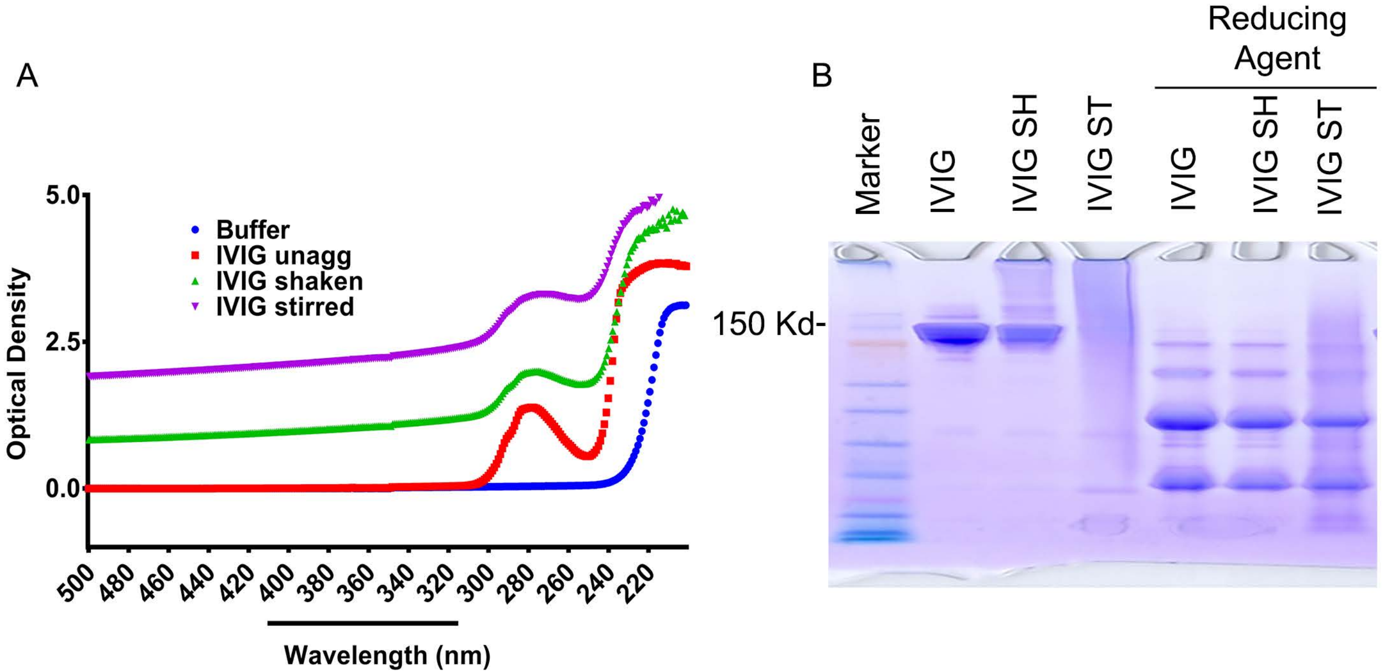

Supplementary Figure 1: Characterization of aggregates IVIG by optical density and SDS-PAGE. Intravenous immunoglobulin IVIG was diluted to 1 mg/mL in sodium citrate buffer (10 mM sodium citrate, 5% sucrose and adjusted the pH to 6.0) stressed under shaken (450 rpm at RT) and stirred conditions (with Teflon stirrer at 1100 rpm at RT. A) The optical density of aggregated protein or unaggregated proteins IVIG was measured (200-500 nm) by using Carry 100UV.VIS spectrophotometer. B) Aggregated and non-aggregated proteins (10 µg) were loaded on SDS-PAGE in the presence or absence of reducing agent, after heated at 100°C for 5 minutes. Electrophoresis was carried out and gels were stained with Coomassie Brilliant Blue. Figure representative of 4 experiments with similar results. Aggregates were stable for a month at 4C.

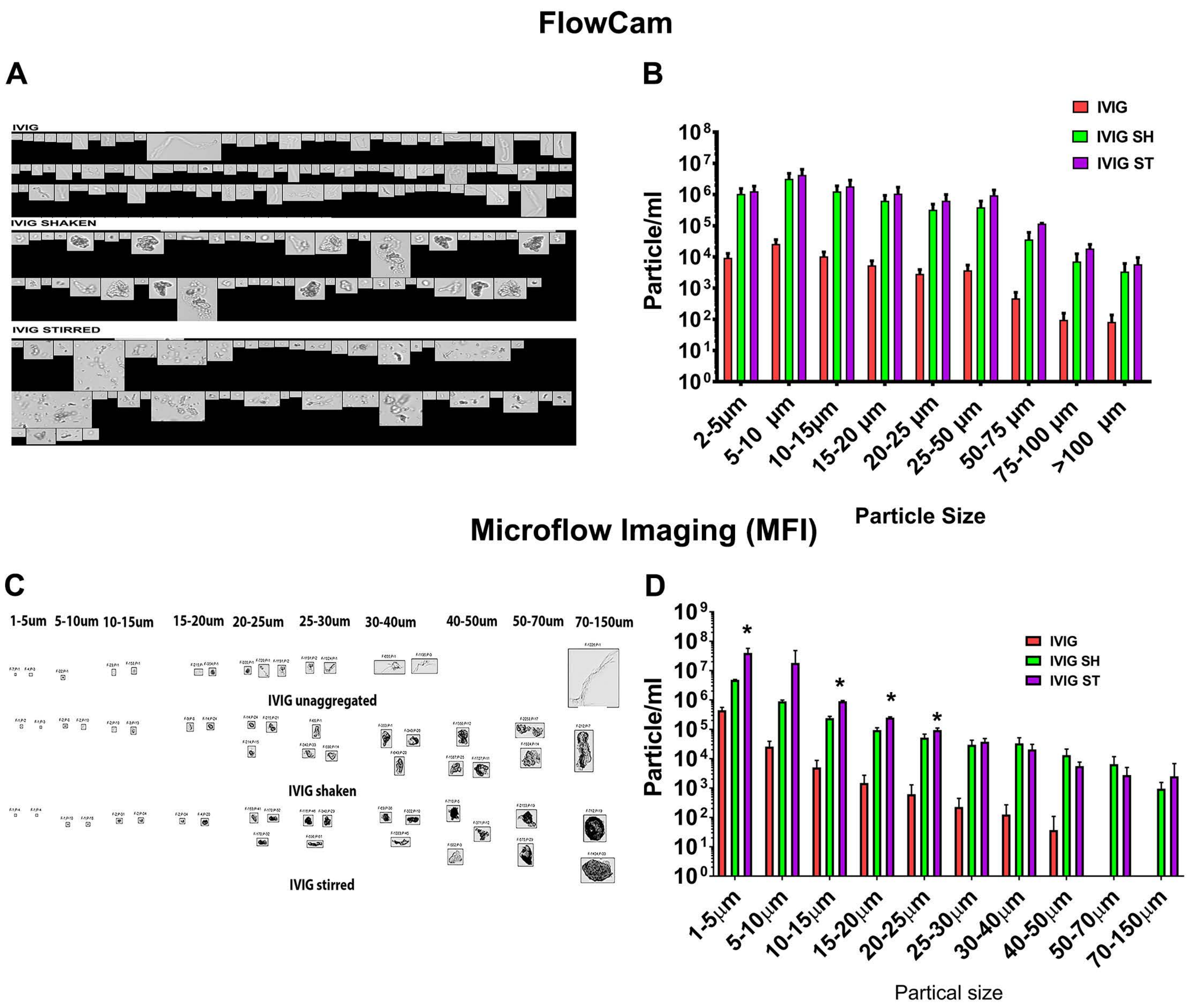

Supplementary Figure 2: Size and morphology of protein particles generated by shaking and stirring of IVIG. Intravenous immunoglobulin IVIG was diluted to1 mg/mL in sodium citrate buffer (10 mM sodium citrate, 5% sucrose and adjusted the pH to 6.0) stressed under shaken (450 rpm at RT) and stirred conditions (with Teflon stirrer at 1100rpm at RT. IVIG aggregates were made the morphology of aggregated proteins were analyzed by FlowCam and MFI. (A) Shows representative images obtained by FlowCam. Results are representative of 2 independent experiments. (B) shows the particle number and size distribution in aggregated and unaggregated IVIG by measured by FlowCam. The trends observed for the particle distribution size for shaken and stirred product are similar when assessed by FlowCam (C&D) Representative particle images (C) and the particle sizes and distribution (D) in aggregated IVIG as assessed by MFI. With MFI there is increased particles in the <10 µm range with the product aggregated using stirring. (B&D) are plotted on a logarithmic scale to allow better visualization of the particles present in the starting material as well as the large numbers generated by the treatment. Results are representative of 4 independent experiments.

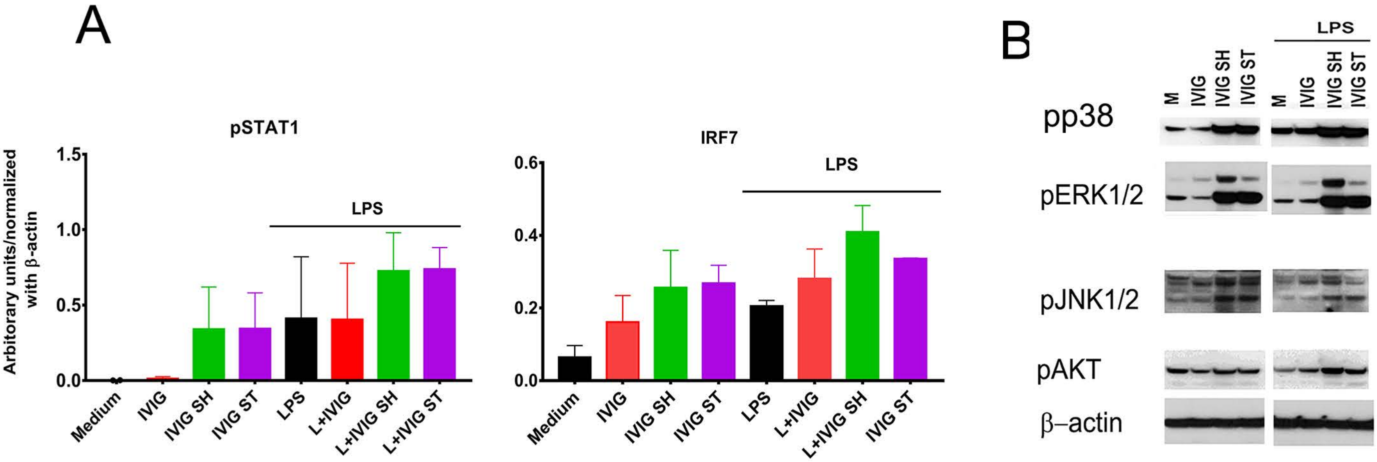

Supplementary Figure 3: Transcription factors induced by aggregates and model IIRMI: Quantitation of STAT1 phosphorylation and IRF7 expression as measured in whole cell lysates of PBMC stimulated with unaggregated or aggregated IVIG in the presence or absence of LPS (100 pg/mL for 12 h) by Western blot. Results show the mean and SEM of 2 independent experiments with healthy blood donors (see Figure 5 for raw data image). B) pp38, pERK, pJNK and pAKT phosphorylation. Whole cell lysates were prepared from Human PBMC after stimulation with aggregate or unaggregated protein in the presence or absence of LPS (100 ng/mL) for 30 minutes and subjected Western analysis using anti-phospho antibodies for pp38, pERK, pJNK and pAKT. Unstimulated cells were used as negative controls (denoted as M).

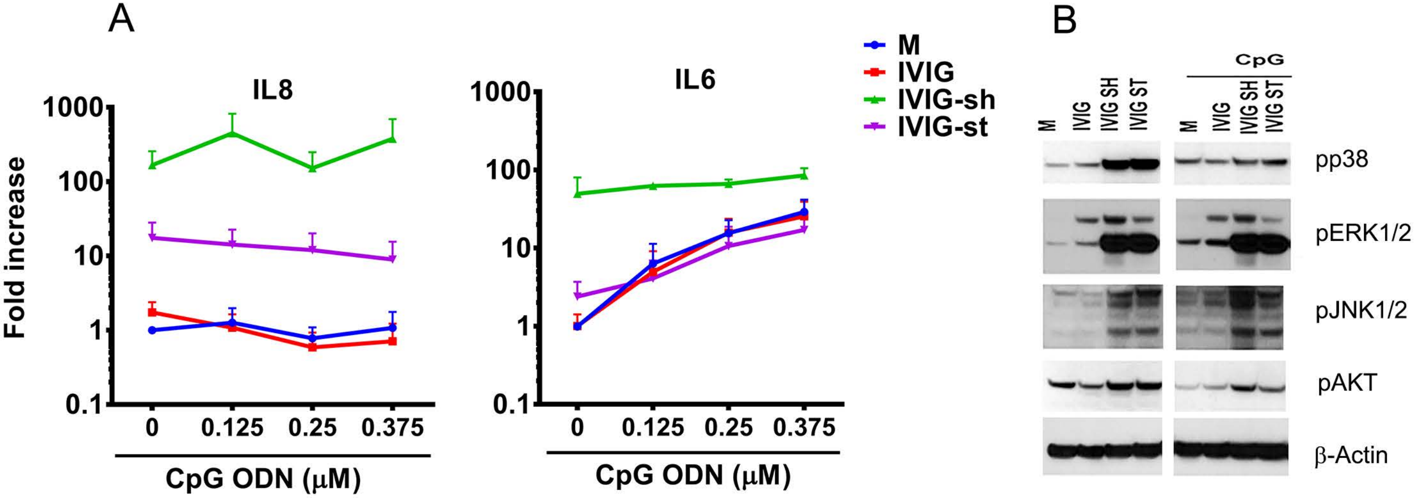

Supplementary figure 4: Aggregates of IVIG do not block the NFκB-dependent pro-inflammatory response to CpG ODN. A) mRNA of IL8 and IL6 were measured by real time PCR following stimulated with aggregated or unaggregated IVIG (80 μg/mL) in the presence or absence of increasing concentration of CpG (D-35) (0.125 μM to 0.375 μM) in human PBMC. B) pp38, pERK, pJNK and pAKT phosphorylation. Whole cell lysates were prepared from Human PBMC after stimulation with aggregate or unaggregated protein in the presence or absence of CpG ODN (0.375 μM) for 30 minutes and subjected Western analysis using anti-phospho antibodies for pp38, pERK, pJNK and pAKT. Unstimulated cells were used as negative controls (denoted as M).

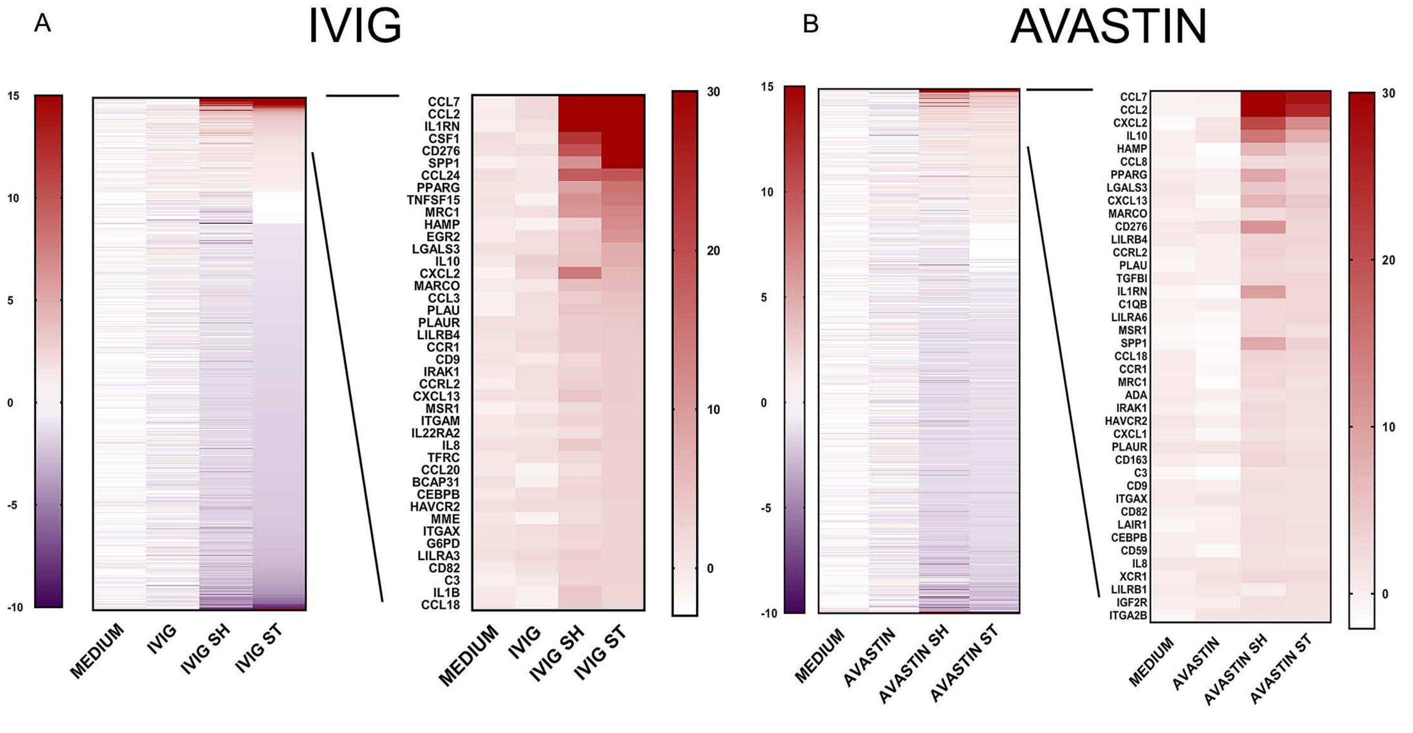

Supplementary Figure 5: Mean RNA expression in PBMC stimulated with intact or aggregated IVIG or Avastin (80 µg/mL; n=6) for 24h as assessed using the Human Immunology V2 Panel (NanoString nCounter). Figure shows A) IVIG and B) Avastin. Panel on the right shows the 40 genes that are more strongly upregulated by the aggregated product. Note that 77% of these genes are common for aggregated IVIG and Avastin. Unstimulated cells were used as negative controls (denoted as M).
